# Functional connectivity of amygdala subnuclei in PTSD: a narrative review

**DOI:** 10.1038/s41380-023-02291-w

**Published:** 2023-10-16

**Authors:** Elizabeth M. Haris, Richard A. Bryant, Thomas Williamson, Mayuresh S. Korgaonkar

**Affiliations:** 1 School of Psychology, University of New South Wales, Sydney, NSW Australia; 2 Brain Dynamics Centre, Westmead Institute for Medical Research, The University of Sydney, Westmead, NSW Australia; 3 Discipline of Psychiatry, Sydney Medical School, Westmead, NSW Australia; 4 Western Sydney Local Health District, Westmead, NSW Australia

**Keywords:** Neuroscience, Diagnostic markers, Psychiatric disorders

## Abstract

While the amygdala is often implicated in the neurobiology of posttraumatic stress disorder (PTSD), the pattern of results remains mixed. One reason for this may be the heterogeneity of amygdala subnuclei and their functional connections. This review used PRISMA guidelines to synthesize research exploring the functional connectivity of three primary amygdala subnuclei, basolateral (BLA), centromedial (CMA), and superficial nuclei (SFA), in PTSD (*N* = 331) relative to trauma-exposed (*N* = 155) and non-trauma-exposed controls (*N* = 210). Although studies were limited (*N* = 11), preliminary evidence suggests that in PTSD compared to trauma-exposed controls, the BLA shows greater connectivity with the dorsal anterior cingulate, an area involved in salience detection. In PTSD compared to non-trauma-exposed controls, the BLA shows greater connectivity with the middle frontal gyrus, an area involved in attention. No other connections were replicated across studies. A secondary aim of this review was to outline the limitations of this field to better shape future research. Importantly, the results from this review indicate the need to consider potential mediators of amygdala subnuclei connectivity, such as trauma type and sex, when conducting such studies. They also highlight the need to be aware of the limited inferences we can make with such small samples that investigate small subcortical structures on low field strength magnetic resonance imaging scanners. Collectively, this review demonstrates the importance of exploring the differential connectivity of amygdala subnuclei to understand the pathophysiology of PTSD and stresses the need for future research to harness the strength of ultra-high field imaging to gain a more sensitive picture of the neural connectivity underlying PTSD.

## Introduction

### The substructures of the amygdala

The amygdala is located deep in the medial temporal lobe and spans a widely distributed network of brain regions. The structure is an integral part of the neural circuitry of emotion regulation [1, 2], and implicated in the pathophysiology of numerous mental health disorders, including anxiety, depression, and posttraumatic stress disorder (PTSD [3, 4]). Although functional magnetic resonance imaging (fMRI) is sensitive to imaging subcortical structures, those in the medial temporal lobe remain: prone to neural signal distortions and signal loss due to shared boundaries with air and bone surfaces which distort the magnetic field [5]; and, limited in their spatial resolution when using low MRI field strengths, such as 1.5 T [6]. The pitfalls of current approaches when imaging amygdala subnuclei are nicely discussed in an article by Foster and colleagues [7], limitations which essentially impact the interpretation of the recorded signals. A further specific problem which compounds the interpretation of recorded signals is that the amygdala itself is often studied in humans as a single structure due to its relatively small size (~2 cm^3^ [8–10]). Yet this disregards its composition as a region comprised of multiple structurally and functionally heterogenous subnuclei across species [11, 12]. As analysis methods improve [12], delineation of the amygdala and its connections to the rest of the brain may help us understand the nature of its contribution to different mental health disorders.

Although the amygdala can be divided into at least 13 nuclei in rodents [13] and nine nuclei in humans [12], the majority of research informing neuropsychiatric research delineates the amygdala into three major subregions [11, 14]. The basolateral subnuclei (BLA) is the largest complex and includes four nuclei—accessory-basal, basal, lateral, and paralaminar [15]. It is heavily connected to the cerebral cortex [16, 17] and largely involved in facilitating associative learning of emotional stimuli [16, 18]. The centromedial subnuclei (CMA) comprises two of the smallest nuclei—central and medial [15], and has extensive connections to the brainstem and hypothalamus [19, 20]. It is involved in the generation of behavioral responses to emotional stimuli, particularly motivational salience [21]. The superficial subnuclei (SFA) includes cortical-like nuclei—the anterior-amygdaloid-area, cortical, and cortico-amygdaloid transition [15]—that are involved in social, affective, and olfactory processing [22, 23]. Importantly, the amygdala does not operate alone, but functions within a complex neural system involving several interconnected brain regions that subserve social [24], affective [25], and motivational functions [21, 26]. Consistent with its crucial role in fear memory and the modulation of fear responses [27], the amygdala has been posited as a key neural region in PTSD—a disorder primarily characterized by impaired fear processes [28].

### The role of the amygdala in PTSD

Posttraumatic stress disorder is a potentially debilitating psychiatric disorder that affects up to 4% of the population, with risk varying dependent on country, type of trauma, and sex sampled [29]. At its core, PTSD is a pathological manifestation of enhanced fear and avoidance responses to traumatic stimuli [30, 31], however, it is also characterized by symptoms of re-experiencing memories, negative cognitions and mood, and dysregulated arousal and reactivity [32]. Decades of research has demonstrated that the amygdala is a crucial component of PTSD [33], primarily due to its role in the acquisition and encoding of fear memories [34].

The prevailing theory underlying the acquisition of fear memory involves fear conditioning at the time of trauma exposure, when stimuli associated with fear responses are encoded in the lateral nucleus of the amygdala; this has been observed in both animals and humans [35]. This conditioning leads to the elicitation of fear responses in rodents via projections from the lateral to the central nucleus of the amygdala [36]. Additionally, the medial prefrontal cortex (PFC) also plays a key regulatory role in fear learning and memory through bidirectional connections with the amygdala in animals and humans [37, 38]. In PTSD, deficient recruitment of the mPFC impedes new learning required for inhibition of initial fear conditioning [39]. Evidence from resting-state and task-based neuroimaging studies in humans also implicates the amygdala as consistently hyperactive in PTSD but only relative to non-trauma-exposed controls [40–42]. Meta-analyses of neuroimaging studies in PTSD relative to trauma-exposed controls instead show both amygdala hyperreactivity [43, 44] and no difference in amygdala activity between groups [41]. Major contributors to these mixed results likely include small sample sizes (which might prevent detection of amygdala activity), the use of cross-sectional study designs (because of varying developmental trajectories of the amygdala since trauma exposure), sex (due to differences in amygdala response to trauma [45]), and trauma type (which may result in heterogenous amygdala responses to trauma [46, 47]).

Furthermore, the nature of the comparison group—trauma-exposed or non-trauma-exposed controls—also mediates the relative morphometry and functionality of the PTSD brain [41, 48–50]. This suggests that trauma exposure alone can result in brain alterations like those seen in PTSD, but that the disorder also demonstrates distinct neurobiological differences. Specifically, meta-analyses have shown decreased resting-state functional connectivity: within the default mode network (DMN) in PTSD relative to both trauma-exposed and non-trauma-exposed controls [40, 51]; between the right superior frontal gyrus and the affective network (which includes the amygdala) in PTSD vs trauma-exposed controls [51, 52]; and, between the affective network and the left middle temporal gyrus in PTSD vs non-trauma-exposed controls [51, 53]. In contrast, hyperconnectivity in PTSD vs trauma-exposed controls was found between the affective network and the right posterior putamen and right dorsolateral PFC [51], which are part of the somatomotor network [54] and frontoparietal attention network [55], respectively. Hyperconnectivity in PTSD relative to both trauma-exposed and non-trauma-exposed controls was also found between the amygdala and insula [51], areas involved in determining stimuli salience [40], that may contribute to the overall attentional processing deficits reported in PTSD [56]. Finally, a meta-analysis by Koch and colleagues [40] showed overall conflicting evidence for dysregulated connectivity in PTSD relative to trauma-exposed controls between the amygdala and PFC (which showed greater connectivity and no difference in connectivity); and, the amygdala and dorsal anterior cingulate cortex (ACC; which showed increased, decreased, and no difference in connectivity). While these results reinforce the importance of the amygdala to the underlying neural dysregulation of the brain in PTSD, they also indicate a lack of clarity in the overall functional characterization of the amygdala in PTSD.

The differential functions of the BLA, CMA, and SFA—the three typical divisions of the amygdala—have also been shown to be impaired in PTSD. Behaviorally, PTSD has been implicated in associative learning deficits of emotional information (i.e., not just fear [57]), particularly in the generalization of past learning to novel situations and extinction of fear-related memories [58, 59]. Individuals with PTSD also demonstrate biases in attention processing that counterintuitively manifests in lower reaction times to trauma-related (i.e., salient) stimuli [60]—perhaps a result of impaired interference or inhibitory processes [60, 61]. Additionally, those with PTSD also show deficits in social and affective cognition, mentalizing, and facial emotion recognition [62], as well as dysfunctional olfactory processing [63, 64]. With evidence of impairments across these differential functions, it is reasonable to assume that amygdala subnuclei may have differential effects on the underlying pathophysiology of PTSD.

Although it is established that the amygdala is important in the pathophysiology of PTSD, and that there are differences in the neural connections of the BLA, CMA, and SFA, the extent of and collective findings from research on amygdala subnuclei in PTSD remains unknown. Accordingly, fine-grained elucidation of the neural connectivity of amygdala subnuclei will aid in further understanding the neural dysfunction underlying PTSD. Furthermore, outlining the effect of limitations of such research, will allow the field to take earlier steps to refine the questions we ask and to gather more accurate data to better answer these questions. Therefore, we conducted a systematic review on studies investigating the neural connectivity of amygdala subnuclei in PTSD. The aim of the review was to elucidate the connectivity profiles of amygdala subnuclei in those with PTSD versus those without the disorder, to outline research findings to date, and to summarize key considerations to inform future research. Considering that previous neuroimaging studies have found differential neural activations in PTSD that are dependent on the nature of the control group [41, 48–50], we decided to analyze PTSD results separately in comparison to both groups. Based on these activation studies, we hypothesized greater connectivity in PTSD between the amygdala and subgenual anterior cingulate, precuneus, postcentral gyrus, and middle frontal gyrus relative to trauma-exposed individuals. We also hypothesized lesser connectivity in PTSD relative to non-trauma-exposed controls between the amygdala and inferior frontal and middle frontal gyri.

## Materials and methods

### Search strategy

The initial literature search included all articles investigating amygdala subnuclei functional connectivity. Studies listed as published or ‘in-press’ and available in English published from inception to January 1, 2021, from the following databases were included: Embase, Ovid, PsycINFO, PubMed, Scopus, and Web of Science. The project was prospectively registered on Prospero (CRD42021226335). A final literature search was conducted in January 2023 for articles published in 2021-22; only one new article from 2022 was found.

**Fig. 1:**
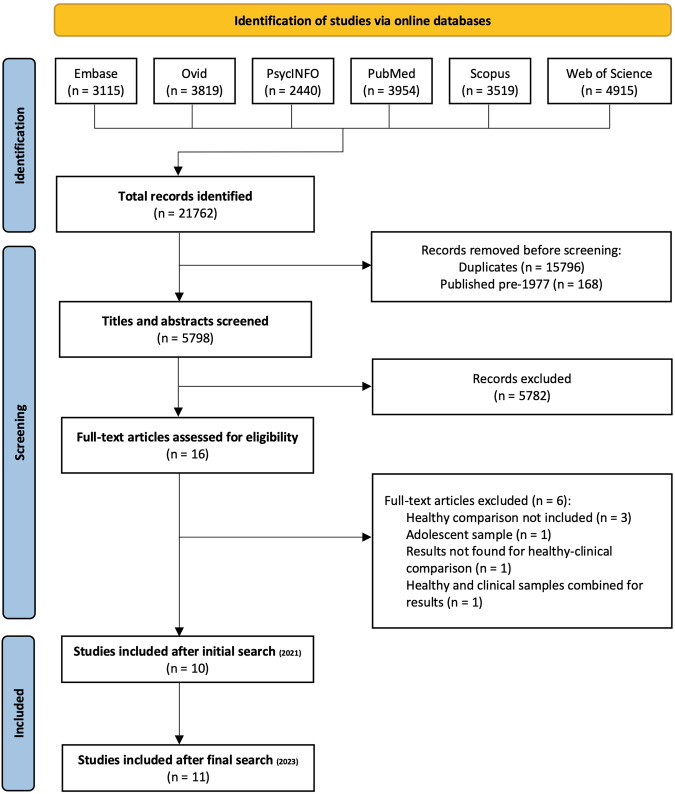
PRISMA [111] flowchart depicting study selection, inclusion, and exclusion.

Title, abstract, and keyword searches were conducted on the amygdala (“amygdala” OR “amygdal*”), and then on terms encompassing imaging connectivity measures and amygdala subregions. Asterisks were used to allow for permutations of all terms (e.g., connect* = connectivity, connectome, connection, etc.). Specific connectivity search terms included “connectivity”, “circuitry”, “covariance”, “projectome”, “pathway”, and were linked using the “OR” Boolean term. Likewise, the “OR” term also linked search terms representing amygdala subnuclei (“amygdala”, “basolateral”, “laterobasal”, “centromedial”, “mediocentral”, “superficial”, “basal”, “lateral”, “cortical”, “central”, “paralaminar”, “medial”, “intercalated”, “extended”, “accessory-basal”, “corticoamygdala”, “cortico-amygdala”, “cortical-amygdala”, “anterior amygdala”, “anterior-amygdala”, “periamygdala”, “bed nucleus of the accessory olfactory tract”, “amygdalo-hippocampal”). Results from these three searches were then linked using Boolean term “AND”. Following this, animal studies were excluded using the “NOT” term to link all database specific subject headings pertaining to animals (e.g., animal, rodent, rat) and remove them from the previous searches. This procedure yielded 5798 studies after duplicates were removed (Fig. 1). Studies prior to 1977 were also removed, as this is when the first whole body image of a human was obtained [65]. Abstracts were screened by two researchers (EMH, TW) using Abstrackr (http://abstrackr.cebm.brown.edu/ [66]), and conflicts were discussed and mediated by a third researcher (MSK). Excluded articles were those investigating developmental populations (due to differing brain networks with adults) and diffusion related connectivity (which measures anatomical connectivity). Though animal studies can inform our understanding of mechanisms underlying PTSD, they are often biased towards the examination of fear conditioning and extinction paradigms. As such, it has become natural to conceptually associate PTSD with fear conditioning—especially when the amygdala is concerned [67, 68]. Additionally, it is difficult to determine if what we are modeling in animals is a representation of the behavior we see in humans with PTSD and it may even be impossible to model in animals some of the PTSD symptoms manifest in humans (e.g., intrusions, flashbacks [67, 68]). We therefore decided to exclude animal studies and confine our review to amygdala subnuclei connectivity in humans with PTSD to better understand their differential connections, contribution, and relevance to the aspects of PTSD that manifest in humans. Full text screening was completed by one researcher (EMH), and data extraction included author, title, age, sex, imaging methodology/tasks used, seeds/target brain areas, atlas used, contrast/s, multiple comparison correction used, and main corrected connectivity results. Whole-brain and seed-based analyses that used Bayesian methods, family-wise error, false discovery rate, or AlphaSim corrections of *p* ≤ 0.05 were considered. References of included articles were checked for inclusion of any relevant studies missed during screening (none were found). Authors Neumeister and Zhu were also (successfully) contacted for further clarification regarding the regions reported in their studies.

### Analysis strategy

Results were extracted for PTSD connectivity greater than and/or less than trauma-exposed controls or non-trauma-exposed controls. Some studies included separate samples of PTSD individuals with and without dissociative symptoms—those with dissociative symptoms were excluded due to evidence of differing brain connectivity to samples without dissociative symptoms [69]. For studies involving pre-post-treatment measures, only pre-treatment group differences were extracted. Areas of connectivity were reported as recorded in original studies.

## Results

### Description of studies

Eleven total studies were found to examine amygdala subnuclei connectivity in PTSD relative to those without PTSD (Table 1). This resulted in 696 participants (trauma-exposed = 155; non-trauma-exposed = 210; PTSD = 331). Three studies by Nicholson et al. [70–72] included overlapping samples (an extra 18.2% of PTSD participants were analyzed in the second functional connectivity study, while an extra 23.1% and 29.0% of non-trauma-exposed controls and PTSD participants, respectively, were analyzed in the third, dynamic causal modeling [DCM] study); as did two studies by Zhu et al. (42% of the PTSD group in the latter study were also analyzed in the first study [73, 74]). Six studies compared PTSD to non-trauma-exposed controls, four studies compared PTSD to trauma-exposed controls, and one study compared PTSD to both groups. All studies only included adults over 18 years of age, with the average age of participants 37.7 years (trauma-exposed = 40.7; non-trauma-exposed = 34.8; PTSD = 37.7; total age range = 26.3–49.9 years). Females comprised 65% of participants. Some participants with PTSD had comorbid anxiety (11.2%) or depression (28.1%). Participants diagnosed with PTSD experienced a range of trauma types, including childhood interpersonal trauma, natural disaster, adult interpersonal violence, or other adult traumatic events.

**Table 1:** Demographics of reviewed articles.

Study	Year	Country	Sample	*N*	Age	Sex	Trauma type	Comorbidities
					M (SD)	(Female %)		
[75]	2014	USA	TEC	22	44 (8.90)	27%	Veterans	NA
			PTSD	20	44.1 (11.0)	20%	Veterans	MDD
[76]	2016	The Netherlands	TEC	40	M: 41.4 (10.6) F: 38.7 (9.48)	50%	Police-related events, childhood trauma	NA
			PTSD	37	M: 42.3 (9.83) F: 37.6 (9.78)	43%	Police-related events, childhood trauma	MDD, dysthymia, panic disorder, specific phobia
[77]	2022	Brazil	NEC	10	35.1 (11.5)	90%	NA	NA
			PTSD	10	35.1 (11.2)	90%	Adult trauma	MDD
[78]	2021	China	NEC	30	48.4 (10.3)	74%	NA	NA
			TEC	33	48.5 (7.50)	79%	Natural disaster	NA
			PTSD	27	49.9 (6.10)	77%	Natural disaster	MDD, anxiety
[79]	2016	Germany	NEC	18	26.3 (8.83)	100%	NA	NA
			PTSD	18	26.6 (5.78)	100%	Interpersonal violence	Pain disorder, anorexia nervosa, social anxiety disorder, recurrent mild depression
[70]	2015	Canada	NEC	40	32.3 (11.4)	73%	NA	NA
			PTSD	36	37.0 (12.7)	85%	Childhood trauma, adult trauma	MDD, panic disorder
[71]^*^	2016	Canada	NEC	40	32.3 (11.4)	73%	NA	NA
			PTSD	44	38 (13.5)	88%	Childhood trauma, adult trauma	MDD, panic disorder, somatoform disorder
[72]^**^	2017	Canada	NEC	52	35.0 (11.5)	71%	NA	NA
			PTSD	62	40.7 (13.4)	81%	Childhood trauma, adult trauma	MDD, OCD, GAD, panic disorder/agoraphobia, social phobia
[80]	2016	Canada	NEC	20	32.5 (11.6)	50%	NA	NA
			PTSD	26	38.8 (12.2)	47%	Childhood trauma, adult trauma	MDD, OCD, panic disorder without agoraphobia, social phobia, specific phobia, eating disorders, somatoform disorder
[73]^***^	2017	USA	TEC	34	35.1 (10.6)	68%	Adult trauma	NA
			PTSD	27	36.0 (9.10)	56%	Adult trauma	NA
[74]	2018	USA	TEC	26	35.3 (10.4)	73%	Adult trauma	NA
			PTSD	24	35.4 (8.90)	71%	Adult trauma	MDD, panic disorder, social phobia, specific phobia

*TEC* trauma-exposed controls, *PTSD* posttraumatic stress disorder, *NEC* non-trauma-exposed controls, *MDD* major depressive disorder, *OCD* obsessive-compulsive disorder, *GAD* general anxiety disorder.

*Study includes the same participants as [70] and additional eight PTSD.

**Study includes the same participants from [71] and additional 12 NEC and 18 PTSD.

***Study includes 10 PTSD participants from [74].

Eight studies used resting-state fMRI (rs-fMRI [70, 71, 73–78]) and two studies used task-based fMRI (tasks described below [79, 80]). Eight studies used a 3 T scanner and two studies used a 1.5 T scanner [73, 74]. Studies only reported results for three broad subregions of the amygdala (BLA, CMA, SFA). Nine studies used functional connectivity as their analytic measure while one resting-state study used DCM to investigate effective connectivity of amygdala subregions with the ventromedial PFC and periaqueductal gray [72]. Though functional and effective connectivity measure neural integration distinctly, we chose to include this paper on effective connectivity in our review of functional connectivity studies as both methods represent the functional organization of neural circuits and can give insight into the association between spatially distinct brain regions (unlike structural measures which represent actual anatomical connections [81, 82]). Only two resting-state studies were treatment-based: one investigated the neurobiological effects of intranasal oxytocin [76] while the other examined the effects of exposure-based therapy on amygdala subnuclei connectivity in PTSD [74]. Two studies used psychophysiological interaction analyses of task-based fMRI to measure task-specific connectivity changes. Neumeister et al. [79] used an event-related design consisting of passive and conscious viewing of trauma-related and neutral images. Rabellino et al. [80] used a block design to measure brain activity while participants passively viewed trauma-related words (i.e., words they had provided that elicited a strong and stressful reaction) and neutral words (i.e., positive or negative words they had provided that did not elicit a strong reaction) presented consciously and subconsciously.

**Fig. 2:**
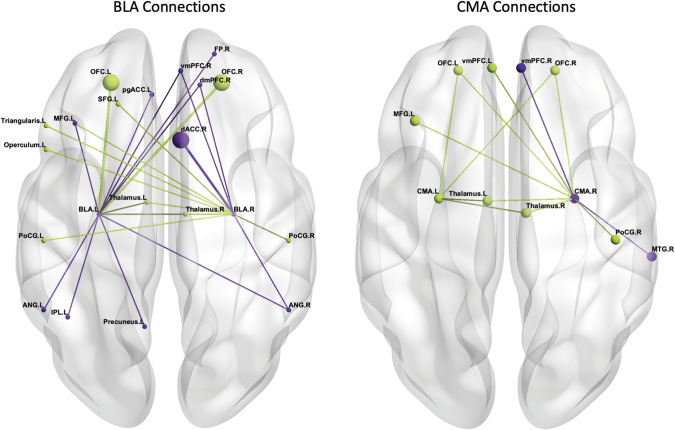
Resting-state amygdala subnuclei connectivity in PTSD vs. trauma-exposed controls. Basolateral subnuclei connections on the left, centromedial subnuclei connections on the right. Greater connectivity for PTSD vs. TEC is signified by dark/purple color; lesser connectivity for PTSD vs. TEC is signified by light/green colors. Image orientation is neurological—left hemisphere on the left, frontal lobe at the top. Larger nodes and edges indicate connections found across multiple studies. PTSD Posttraumatic stress disorder, TEC trauma-exposed controls, L left, R right, BLA basolateral amygdala nucleus, CMA centromedial amygdala nucleus, FP frontal pole, M/SFG middle/superior frontal gyrus, pg/dACC perigenual/dorsal anterior cingulate cortex, OFC orbitofrontal cortex, vm/dmPFC ventro/dorsomedial prefrontal cortex, M/STG middle/superior temporal gyrus, PoCG postcentral gyrus, IPL inferior parietal lobe, ANG angular gyrus.

**Fig. 3:**
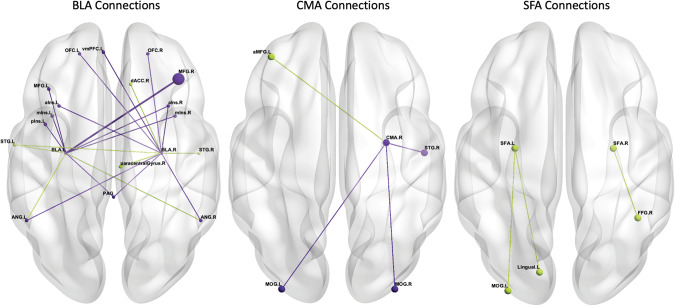
Resting-state amygdala subnuclei connectivity in PTSD vs. non-trauma-exposed controls. This text is supposed to go below the image, as a caption -->Basolateral subnuclei connections on the left, centromedial subnuclei connections in the middle, superficial subnuclei connections on the right. Greater connectivity in PTSD vs. NEC participants is signified by dark/purple colors; lesser connectivity for PTSD vs. NEC is signified by light/green colors; SFA connections show only lesser connectivity for PTSD vs NEC. Image orientation is neurological—left hemisphere on the left, frontal lobe at the top. Larger nodes and edges indicate connections found across multiple studies. PTSD Posttraumatic stress disorder, NEC Non-trauma-exposed controls, L left, R right, BLA basolateral amygdala nucleus, CMA centromedial amygdala nucleus, SFA superficial amygdala nucleus, a/MFG anterior/middle frontal gyrus, dACC dorsal anterior cingulate cortex, OFC orbitofrontal cortex, vmPFC ventromedial prefrontal cortex, a/m/pIns anterior/mid/posterior insula, FFG fusiform gyrus, STG superior temporal gyrus, ANG angular gyrus, MOG middle occipital lobe, PAG periaqueductal gray.

An overview of studies can be found in Table 2. Results for all studies can be found in Table 3. For ease of interpretation, results have been reported in tables and figures comparing resting-state connectivity of amygdala subnuclei between PTSD and trauma-exposed controls (Supplementary Table 1, Fig. 2) and PTSD and non-trauma-exposed controls (Supplementary Table 2, Fig. 3), and those comparing task-based connectivity (Supplementary Table 3, Supplementary Fig. 1).

**Table 2:** Description of reviewed articles.

Study	Comparison group	Imaging methodology		Brain areas analyzed			Amygdala Atlas	Statistical correction
		Scanner/paradigm	Length of paradigm	Seed	Target	Whole brain		
[75]	TEC	3T rs-fMRI	6 min 30 s	Bilateral BLA Bilateral CMA	-	x	Juelich Brain Atlas	Gaussian Random Field Theory *p*<0.05
[76]	TEC	3T rs-fMRI	7 min 54 s	Bilateral BLA Bilateral CMA	Bilateral vmPFC/scACC, bilateral dmPFC/superior frontal gyrus, bilateral middle frontal gyrus, bilateral OFC, bilateral ACC/paracingulate gyrus, bilateral insula	-	Juelich Brain Atlas	*p*_FWE_<0.05, Bonferroni correction *p*<0.0063
[77]	NEC	3T rs-fMRI	7 min	Bilateral BLA Bilateral CMA Bilateral SFA	-	x	Juelich Brain Atlas	3dClustSim *p*<0.005 k = 44
[78]	NEC, TEC	3T rs-fMRI	8 min 28 s	Bilateral BLA Bilateral CMA	-	x	Juelich Brain Atlas	AlphaSim corrected *p*<0.05, Uncorrected *p*<0.001 k = 10
[79]	NEC	3T task-based MRI (passive viewing of trauma-related/neutral images)	8 min 19 s	Left BLA	Bilateral vmPFC, bilateral dmPFC, bilateral vACC, bilateral dACC, bilateral insula, bilateral thalamus, bilateral hippocampus, bilateral occipital area, bilateral brainstem	x	SPM Anatomy Toolbox	Uncorrected *p*<0.005, Monte Carlo simulation (1000) with cluster level false positive rate of 1.67%
[70]^*^	NEC	3T rs-fMRI	6 min	Bilateral BLA Bilateral CMA	Bilateral dlPFC	x	SPM Anatomy Toolbox	*p*_FWE_<0.05 k = 10, Uncorrected *p*<0.05 k = 10
[71]^*^	NEC	3T rs-fMRI	6 min	Bilateral anterior insula Bilateral mid insula Bilateral posterior insula	Bilateral SFA Bilateral BLA Bilateral CMA	-	MNI PickAtlas	*p*_FWE_<0.05 k = 10
[72]^*^	NEC	3T rs-fMRI	6 min	Bilateral BLA Bilateral CMA	Bilateral vmPFC, bilateral periaqueductal gray	-	SPM Anatomy Toolbox	(DCM) Models with an exceedance probability of >0.8 (*p*<0.001)
[80]	NEC	3T task-based MRI (passive viewing of trauma-related/neutral words)	9 min 6 s	Bilateral BLA Bilateral CMA	Right mPFC, bilateral superior colliculus, left pulvinar	x	SPM Anatomy Toolbox	*p*_FWE_<0.05, *p*_FWE_<0.004
[73]^**^	TEC	1.5T rs-fMRI	5 min	Bilateral BLA Bilateral CMA	Bilateral OFC, bilateral sgACC, bilateral thalamus, bilateral hippocampus, bilateral nucleus accumbens	x	Juelich Brain Atlas	*p*_FDR_<0.005 k = 20, Bonferroni *p*<0.012
[74]^**^	TEC	1.5T rs-fMRI	5 min	Bilateral BLA Bilateral CMA	Bilateral mPFC, bilateral OFC Bilateral ACC, bilateral subcallosal cortex, bilateral insula, bilateral thalamus	x	Harvard-Oxford Atlas	*p*_FWE_<0.05 k = 20 voxels, Bonferroni *p*<0.002

*PTSD* posttraumatic stress disorder, *TEC* trauma-exposed controls, *NEC* non-trauma-exposed controls, *DCM* dynamic causal modeling, *BLA* basolateral amygdala nucleus, *CMA* centromedial amygdala nucleus, *SFA* superficial amygdala nucleus, *m/vm/dm/dlPFC* medial/ventromedial/dorsomedial/ dorsolateral prefrontal cortex, *v/d/sg/scACC* ventral/dorsal/subgenual/subcallosal anterior cingulate cortex, *OFC* orbitofrontal cortex, *FWE* family-wise error correction, *FDR* false discovery rate.

*,**Studies used overlapping samples, further details in the demographics in Table 1.

**Table 3:** Main findings of reviewed articles.

Study	Main connectivity findings			
	PTSD > control group		PTSD < control group	
[75]	PTSD > TEC	Right BLA – right dmPFC, right dACC (right mPFC) Left BLA – left pgACC, right dmPFC, right frontal pole, left precuneus, left inferior parietal lobe	PTSD < TEC	Right BLA – left pars opercularis/left pars triangularis (left inferior frontal gyrus)
[76]	Female PTSD > Female TEC	Right BLA – right dACC	Male PTSD < Male TEC	Right CMA – left vmPFC
[77]	-	-	PTSD < NEC	Right SFA – right fusiform gyrus Left SFA – left lingual gyrus, left middle occipital gyrus
[78]	PTSD > TEC PTSD > NEC	Left BLA – right vmPFC, bilateral angular gyrus, left middle frontal gyrus Right BLA – R vmPFC, bilateral OFC, right angular gyrus Right CMA – right vmPFC, right middle temporal gyrus Left BLA – bilateral angular gyrus, bilateral middle frontal gyrus Right BLA – left vmPFC, bilateral OFC, bilateral angular gyrus Right CMA – right superior temporal gyrus, bilateral middle occipital gyrus	PTSD < TEC PTSD < NEC	Right BLA – bilateral postcentral gyrus, left superior frontal gyrus, left middle frontal gyrus Right CMA – right postcentral gyrus, left middle frontal gyrus Left BLA – left superior temporal gyrus Right BLA – bilateral superior temporal gyrus, right dACC, right paracentral gyrus Right CMA – left anterior middle frontal gyrus
[79]	PTSD > NEC	Trauma-related > Neutral images Left BLA – left dACC/mPFC, right dACC, bilateral middle frontal gyrus, right medial frontal gyrus, bilateral insula, right superior temporal gyrus, right middle temporal gyrus, right inferior temporal gyrus, left inferior parietal lobe, left hippocampus, bilateral Brodmann Area 28, right parahippocampal gyrus, left brainstem.	PTSD < NEC	Trauma-related > Neutral images Left BLA – right Brodmann Area 7, right middle temporal gyrus, right postcentral gyrus.
[70]^*^	PTSD > NEC	Left BLA – right middle frontal gyrus	-	-
[71]^*^	PTSD > NEC	Right BLA – bilateral anterior insula Left BLA – right anterior insula, bilateral mid insula, left posterior insula	-	-
[72]^*^	PTSD > NEC	Bilateral BLA – bilateral periaqueductal gray	-	-
[80]	PTSD > NEC	Subliminal/Supraliminal trauma > Neutral words Right CMA – right superior frontal gyrus Left CMA – left pulvinar	PTSD < NEC	Subliminal trauma > Neutral words Right BLA – right superior colliculus
[73]^**^	-	-	PTSD < TEC	Bilateral BLA – bilateral OFC Bilateral CMA – bilateral thalamus
[74]^**^	-	-	PTSD < TEC	Bilateral BLA – bilateral OFC Bilateral CMA – bilateral OFC Bilateral BLA – bilateral thalamus

*PTSD* posttraumatic stress disorder, *TEC* trauma-exposed controls, *NEC* non-trauma-exposed controls, *BLA* basolateral amygdala nucleus, *CMA* centromedial amygdala nucleus, *SFA* superficial amygdala nucleus, *m/vm/dmPFC* medial/ventromedial/dorsomedial prefrontal cortex, *d/pgACC* dorsal/perigenual anterior cingulate cortex, *OFC* orbitofrontal cortex, *Brodmann Area 7* superior parietal lobe/precuneus, *Brodmann Area 28* entorhinal cortex.

*,**Studies used overlapping samples, further details in the demographics in Table 1.

### Resting-state connectivity

#### PTSD relative to trauma-exposed controls

Two papers reported the same significant connection of greater connectivity in the PTSD group between the right BLA and right dorsal ACC [75, 76], however, one sample was primarily composed of males [75] while the other result was only found in females [76]. Greater connectivity in PTSD was also reported in single studies for the left BLA and right frontal pole, left perigenual anterior cingulate cortex [75], left inferior parietal lobe, left precuneus [75], and bilateral angular gyrus [78]. Liu and colleagues [78] also found significantly greater connectivity for PTSD for *both* the bilateral BLA and right CMA and the right ventromedial PFC. Lesser connectivity in PTSD was reported between the right BLA and left pars triangularis, left pars opercularis [75], left postcentral gyrus, and left superior frontal gyrus [78]. Liu and colleagues [78] also found significantly lesser connectivity for PTSD for *both* the right BLA and right CMA and the left middle frontal gyrus and right postcentral gyrus. Similarly, Zhu and colleagues found lesser connectivity for PTSD for *both* the bilateral BLA [74] and CMA [73] and bilateral thalamus, and for *both* the left BLA [73, 74] and bilateral CMA [74] and bilateral orbitofrontal cortex (OFC).

Single study resting-state connectivity for the CMA was also reported. Greater connectivity for PTSD was reported between the right CMA and right middle temporal gyrus and right ventromedial PFC [78]. In contrast, lesser connectivity was reported for PTSD between the right CMA and left ventromedial PFC [76]. No studies involving trauma-exposed controls investigated the connectivity of the SFA.

#### PTSD relative to non-trauma-exposed controls

Greater resting-state connectivity was reported between the left BLA and right middle frontal gyrus for two studies [70, 78]. Results from single studies reported greater connectivity between the left BLA and right anterior insula, bilateral mid insula, and left posterior insula [71], and bilateral angular gyri [78], and between the right BLA and left ventromedial PFC, left middle frontal gyrus, and bilateral OFC [78], and left anterior insula [71]. The study using DCM [72] reported a directed, top-down connection from the bilateral BLA to the bilateral periaqueductal gray that was unique to PTSD. In contrast, lesser connectivity in PTSD was only found by Liu and colleagues [78] between the left BLA and left superior temporal gyrus, and between the right BLA and bilateral superior temporal gyrus, right paracentral gyrus, and right dorsal ACC.

Of the five studies that also investigated the CMA, only one [78] found significant between-groups differences, specifically, greater connectivity in PTSD between the right CMA and right superior temporal gyrus and bilateral middle occipital gyrus and lesser connectivity between the right CMA and left anterior middle frontal gyrus.

Two studies investigated connectivity of the SFA nucleus, but only one [77] found significant between-groups differences, specifically, lesser connectivity in PTSD between the right SFA and right fusiform gyrus, and between the left SFA and left lingual gyrus and left middle occipital gyrus. Interestingly, they did not find any between-group differences for the other two nuclei.

### Task-based connectivity (non-trauma-exposed controls)

Only two studies investigated task-based connectivity. Neumeister and colleagues [79] examined the passive viewing of trauma-related images vs. neutral images, and reported greater connectivity in PTSD between the left BLA and several frontal areas (bilateral dorsal ACC, bilateral middle frontal gyrus, right medial frontal gyrus, bilateral insula), temporal areas (right inferior temporal gyrus, right superior temporal gyrus, left hippocampus, right parahippocampal gyrus, bilateral Brodmann Area 28 [roughly, the entorhinal cortex]), the left inferior parietal lobe, and left brainstem. Lesser connectivity to trauma-related images in PTSD was reported between the left BLA and right postcentral gyrus and right Brodmann Area 7 (roughly, the precuneus).

Rabellino and colleagues’ [80] study investigated passive viewing of supra- and subliminal words, revealing greater connectivity in PTSD between the right CMA and right superior frontal gyrus and between the left CMA and left pulvinar for both conscious and subconscious viewing of trauma-related words. Lesser connectivity for PTSD was reported between the right BLA and right superior colliculus for the subconscious viewing of trauma-related words.

### Contradictory connections across subregions

Of note, some contradictory connections were found among the included studies. Specifically, Liu and colleagues [78] reported greater resting-state connectivity in PTSD vs. trauma-exposed controls between the right BLA and bilateral OFC, however, Zhu and colleagues’ studies [73, 74] reported lesser resting-state connectivity. Additionally, Neumeister and colleagues’ [79] task-based study reported greater connectivity between the left BLA and right middle temporal gyrus for trauma-related vs. neutral images for *both* PTSD and non-trauma-exposed controls. For clarity, these contradictory connections have not been included in the figures.

## Discussion

This review set out to elucidate the functional connectivity profiles of amygdala subregions in PTSD to better understand the neural dysfunction underlying the disorder and to outline the limitations of this field to refine the accuracy of future research. We did this through the analysis of functional connectivity studies in PTSD relative to non-trauma-exposed and trauma-exposed controls, to account for the moderation of neural profiles by comparison group [41, 48–50]. While these findings should be interpreted with caution due to the limited number of studies in the area, there is no doubt that investigating the connectivity of amygdala subregions in PTSD will allow a more fine-grained elucidation of the nature of their differential connectivity patterns. The limitations of these studies point to the need for more refined research methods, such as ultra-high field imaging, to better understand amygdala subfield connectivity patterns.

### The role of amygdala subnuclei in PTSD

#### Resting-state connectivity in PTSD relative to trauma-exposed controls

Greater connectivity between the right BLA and right dorsal ACC in PTSD was the only significant finding reported across two studies [75, 76]. Rodent studies show that mono- (cortical→subcortical) and bidirectional connections between the BLA and dorsomedial PFC (rodent homolog) modulate avoidance and anxiety behaviors [83, 84]. In non-human primates, cingulate areas 24 and 25 demonstrate high mono-directional connections with the BLA that have been found to support learning rate and memory strength during aversive learning [85, 86]. Studies in healthy humans also show negative associations between bilateral BLA and bilateral dorsal ACC activity [14], which fits with our results—particularly when considering that activity in the ACC switches from ventral to dorsal dominance with stronger threat—and supports the role of the dorsal ACC in salience detection [87]. In one study, however, greater connectivity was found in a largely male sample (80% [75]), while the results from the other study [76] were only found in the females of the sample. While speculative, this may be due to an interaction between trauma type and the neurobiological effect of early life trauma exposure [45]. In a review of sex differences in trauma-related psychopathology, Helpman and colleagues [45] found that childhood trauma exposure in females likely involves an overactive and possibly enlarged amygdala, while in males it likely involves overactivity and increased connectivity of the dorsal ACC. This could account for these disparate findings as the sample of females had experienced both childhood and adult trauma, but the largely male sample was comprised of military veterans who may not have experienced childhood trauma (only lifetime trauma was measured). In support of this interpretation, a study by Engman and colleagues [88] found greater connectivity between the BLA and ACC in healthy women with low levels of estrogen, a hormonal pattern which has been associated with negative affect [89]. They also found that healthy women with higher/lower levels of estrogen show greater connectivity between the CMA/BLA and sensory processing areas. This indicates a potentially higher sensitivity in emotion processing networks in women generally, which supports the higher prevalence of PTSD in females [45]. Taken together, this result illustrates the importance of considering both the comparison group and sex of participants when seeking to clarify the neuropathology of PTSD, and may even indicate the need to look deeper at hormonal fluctuations and trauma type to fully understand the differential connectivity of amygdala subnuclei in PTSD.

When examining the single study results as a whole, a lateralization pattern was found indicating greater connectivity for the PTSD group between the left BLA and left hemisphere parietal regions involved in the DMN (inferior parietal lobe, precuneus, angular gyri) and lesser connectivity between the right BLA and left frontal regions involved in the frontoparietal attention network (pars opercularis, pars triangularis, postcentral gyrus, superior frontal gyrus). Accordingly, PTSD has been linked to lower functional connectivity between regions involved in the DMN, especially the precuneus and angular gyrus [90], which might occur due to interference from and increased connectivity with the BLA, though this will have to be explored in future studies. In addition, combined results for the CMA also show a lateralization pattern, with PTSD demonstrating greater connectivity between the right CMA and right frontal and temporal areas (ventromedial PFC, middle temporal gyrus) and lesser connectivity between right CMA and left frontal regions (ventromedial PFC, middle frontal gyrus). While previous research has shown the right amygdala to be largely associated with fear conditioning [91, 92] and the left amygdala to be associated with negative affect [93], future research into amygdala subnuclei connectivity in PTSD may help us to better understand this dissociable functional pattern.

#### Resting-state connectivity in PTSD relative to non-trauma-exposed controls

Greater connectivity between the left BLA and right middle frontal gyrus in PTSD was the only significant finding reported across two studies [70, 78]. It has been suggested that the right middle frontal gyrus is a brain region where dorsal and ventral attention networks converge, an area that is involved in reorienting attention from endogenous to exogenous stimuli [94, 95] and in conflict resolution [96]. Yet, while neuronal connectivity between the BLA and medial PFC (middle frontal gyrus/dorsolateral PFC homolog) in rodents shows strong reciprocal connections [97], negative associations between the bilateral BLA and middle frontal gyrus in healthy humans at rest have been demonstrated [14]. Greater connectivity between the left BLA and right middle frontal gyrus in PTSD may represent a shift in the typical functioning of this connection which may contribute to the attentional difficulties observed in behavioral studies of PTSD [56]. These results were found in studies with mostly female participants (~80%) with comorbid depression and anxiety disorders, but in those who had experienced different trauma types (natural disaster, various childhood/adult trauma), and were in different parts of the world (China, Canada). This suggests a somewhat robust result, particularly considering that Neumeister et al. [79] also found this connectivity pattern in their task-based study comprised of all females. A recent meta-analysis [51] found hyperconnectivity between affective network seeds (which includes the amygdala) and the dorsolateral PFC (which is encompassed by the middle frontal gyrus) in PTSD relative to all controls in their sample that seemed to be driven by a difference between PTSD and trauma-exposed controls. However, it is possible that this was due to the limited number of studies in the meta-analysis that used amygdala seeds (10 with trauma-exposed comparison and 8 with non-trauma-exposed comparison). Collectively, it appears that there is an association between the BLA and the middle frontal gyrus in PTSD, but more research is needed across both comparison samples to determine the strength of the result on a larger scale.

Single study results did not especially show a discernible pattern. However, greater BLA connectivity in PTSD was evident with frontal areas involved in reward and goal-directed learning and behavior (OFC, ventromedial PFC [98, 99]) and attention (insula, middle frontal gyrus [95, 100]), which is largely consistent with work in primates [86, 101]. While many connections were observed for the BLA, only one of five studies reported significant alterations in connections for the CMA [78], and only one of two studies for the SFA [77]. Nevertheless, these results still demonstrate the importance of investigating amygdala subnuclei connectivity. The right BLA showed lesser connectivity to the right superior temporal gyrus, but the right CMA showed greater connectivity to this area. Similarly, the right CMA showed greater connectivity to the left middle occipital gyrus, but the left SFA showed lesser connectivity with this region. Such responses may be canceled out if examining the connectivity of the amygdala as a homogenous structure, which only reiterates the importance of investigating the differential connectivity of amygdala subnuclei with other brain regions.

#### Task-based connectivity in PTSD relative to non-trauma-exposed controls

Only two studies investigated task-based subnuclei connectivity in PTSD [79, 80]. This makes it difficult to draw any solid conclusions from the results, especially considering the results from these studies did not overlap. Collectively, the BLA appears to show greater connectivity with frontal and temporal areas implicated in higher-order processing. However, although several reviews show hyperactivation of the amygdala, dorsal ACC, and hippocampus in PTSD [41, 43, 44], functional connectivity findings across task-based studies are fewer and remain inconsistent. Specifically, they show greater amygdala-medial PFC connectivity for symptom provocation paradigms [102], but both greater and lesser connectivity between these same areas for fear processing paradigms [49, 103]. This highlights the need for further research in this area to disentangle the functional differences of amygdala subnuclei connectivity in PTSD.

### The limitations of current amygdala subnuclei research in PTSD

#### Sample

One of the most important limitations that must be considered with this review is the nature of the comparison group. Most studies investigating PTSD chose *either* a trauma-exposed or a non-trauma-exposed comparison group; indeed, only one out of the 11 studies included both groups [78]. However, the comparison group mediates any differences found between brain regions, as demonstrated in this paper and reported in previous meta-analyses [41, 48, 49]. Trauma-exposed controls are the ideal comparison group because it cannot be determined if neurobiological differences between PTSD and non-trauma-exposed controls are due to PTSD proper or to trauma exposure in general. However, comparing PTSD to both trauma-exposed and non-trauma-exposed controls can provide a richer picture of the connectivity of amygdala subnuclei in PTSD. For instance, the consistent result seen for PTSD vs. trauma-exposed controls shows greater BLA connectivity with the dorsal ACC, a region involved in the salience network. In contrast, the consistent result demonstrated for PTSD vs. non-trauma-exposed controls shows greater BLA connectivity with the middle frontal gyrus, a region involved in the frontoparietal attention network. Furthermore, Patel and colleagues [41] in their meta-analysis found hyperactivation of the amygdala in PTSD, but only when compared to non-trauma-exposed participants, which supports the idea that amygdala hyperactivation may occur more generally in relation to trauma exposure. Collectively, this highlights a potential spectrum of neurobiological differences between non-trauma-exposed controls, trauma-exposed controls, and individuals with PTSD, in which amygdala subnuclei may play a part.

Other limitations specific to the studies included in this review, include the cross-sectional nature of their designs and small sample sizes. Cross-sectional studies examine data from the population at one point in time. While this can highlight differences between groups, it cannot tell us about whether these differences precede or follow the outcome [104], which must be done using longitudinal studies. However, longitudinal studies in PTSD also suffer from a number of limitations themselves, particularly the practicalities of assessing participants immediately after trauma exposure [105]. Therefore, in using cross-sectional designs we must be cautious about inferring causal relationships based on any group differences we find. Regarding the latter limitation, the average sample sizes of studies in this review consisted of 30 participants per group and ranged from 10 [77] to 62 participants [72]. Such small sample sizes as 10 may not allow the detection of clinically significant differences between groups [104] particularly when investigating small subcortical nuclei, hence results from these studies need to be interpreted with caution. Illustrating this point are the studies by Zhu and colleagues [73, 74], which used slightly overlapping PTSD samples (half the sample was the same), yet only found one overlapping result (bilateral BLA – bilateral OFC connectivity). This may illustrate that the initial result was not robust enough to survive with the addition of more participants, which again indicates that we need to interpret these results cautiously.

#### Methodology

Zhu et al’s. [73, 74] study also suffers from a further, methodological limitation: that of using a scanner with lower field strength (1.5 T) which provides images with lower spatial resolution. Anatomically, the amygdala is a relatively small region susceptible to magnetic field inhomogeneities during neuroimaging due to its location near air-filled cavities near the base of the skull [106]. Therefore, lower resolution scanners may not have adequate signal-to-noise ratio to resolve signals from amygdala subnuclei [107]. This is also true of 3 T scanners when imaging subcortical structures [7, 108], which is likely the reason that most amygdala subnuclei research thus far has not investigated all nine subnuclei but rather combines smaller nuclei into three larger structures. Additionally, standard image pre-processing steps, such as smoothing, further degrade images obtained from a scanner [109], which may widen the area of amygdala activation, and lead to overlapping signals from separate subnuclei. Importantly, these limitations can be rectified in future studies through utilization of ultra-high field imaging and more sensitive data acquisition and analytical techniques [107].

Two further methodological points to be mentioned are that of the subnuclei examined and the analysis methods. While there is a distinct lack of studies in this area, 10 studies examined both the BLA and CMA but only two studies examined the SFA [71, 77]. It is unclear why this is the case, although it could be due to the SFA being primarily linked to olfactory processing rather than the traditional fear and emotional processing with which both the BLA and CMA are associated [22, 27]. Whatever the historical reason, the emerging role of the SFA in social and affective processing [14, 22] is worth further exploration in PTSD. Similarly, while all studies used rs-fMRI to examine the connectivity of amygdala subnuclei, 10 studies used traditional functional connectivity to analyze results while one study used DCM to analyze the data [72]. In contrast to functional connectivity analyses which can examine statistical correlations across the entire brain [81], DCM requires the comparison of models fitted to data using Bayesian statistics which requires hypotheses about which brain regions to examine [110]. Therefore, DCM allows for the interpretation of directed and mechanistic inferences about brain connectivity. However, as mentioned above, both functional connectivity and DCM methods represent the functional organization of neural circuits [81, 82], and we only input the group differences between brain regions found within the DCM study (i.e., the PTSD group showed top-down connectivity of the BLA to the periaqueductal gray which was not shown in controls, while all other connections shown were stronger in PTSD than in controls). As we were interested in trying to establish group differences, the addition of these results doesn’t require any inference about directionality of connectivity. Nevertheless, the methodology of this paper is different from the others, and again, it’s results must be interpreted with caution. Collectively, these limitations point to specific areas of importance that must be considered when conducting research investigating amygdala subnuclei connectivity in PTSD.

## Conclusion

The whole amygdala has consistently been implicated in the pathophysiology of PTSD due to its primary role in fear processes; however, this disregards the heterogeneity of the structure. Traditional research divides the structure into three main regions—the BLA, CMA, and SFA—which all have differential roles. However, the connectivity of these subregions in individuals with PTSD remains unclear. This study sought to elucidate the connectivity of amygdala subnuclei in PTSD and to outline the limitations in this field for the refinement of future research. To that end, 11 studies were found investigating amygdala subnuclei connectivity in PTSD, but only two findings emerged across two studies. Greater connectivity between the right BLA and right dorsal ACC was found in PTSD relative to trauma-exposed controls [75, 76], and greater connectivity was found between the left BLA and right middle frontal gyrus in PTSD relative to non-trauma-exposed controls [70, 78]. The results from this review suggest a potentially important role in PTSD between the BLA and regions involved in salience and attention, also highlighting the potentially different roles of the left and right BLA in PTSD. More pressingly, they point to the potential mediation of neural connectivity based on trauma type and sex of participants, the caution with which we must interpret results due to small sample sizes and use of low-resolution MRI scanners, and the need for the inclusion of all amygdala subnuclei in this research. Though difficult, future studies would benefit from larger and more homogenous samples; taking as many variables into account if this is not possible. Furthermore, harnessing the strength of ultra-high field MRI scanners and cutting-edge analysis methods is imperative to truly gain an understanding of the differential connectivity profiles of amygdala subnuclei in PTSD.

### Supplementary information

Supplementary Information
