# Supplementary material for: Functional connectivity of amygdala subnuclei in PTSD: a narrative review
Source: Mol Psychiatry. 2023 Oct 16;28(9):3581–94. doi: 10.1038/s41380-023-02291-w (PMC10730419; doi:10.1038/s41380-023-02291-w)
Supplement: Supplementary file 1 — Supplementary Information [file 41380_2023_2291_MOESM1_ESM.docx]

**Supplementary Information**

**Supplementary Table 1.**

*Table of Main Resting-State Functional Connectivity Results for Posttraumatic Stress Disorder (PTSD) vs. Trauma-Exposed Healthy Controls (TEC)*

| **Contrast** | **Brain Region** | **BLA** | |  | **CMA** | |  | **Contrast** | **Brain Region** | **BLA** | |  | **CMA** | |
| --- | --- | --- | --- | --- | --- | --- | --- | --- | --- | --- | --- | --- | --- | --- |
|  |  | L | R |  | L | R |  |  |  | L | R |  | L | R |
| PTSD > TEC |  |  |  |  |  |  |  | PTSD < TEC |  |  |  |  |  |  |
| *Frontal* |  |  |  |  |  |  |  | *Frontal* |  |  |  |  |  |  |
|  | Right vmPFC | x^1^ | X^1^ |  |  | x^1^ |  |  | Left vmPFC |  |  |  |  | x |
|  | Left Orbitofrontal Cortex |  | x^2^ |  |  |  |  |  | Left Orbitofrontal Cortex | x^1^ | x^2^ |  | x^1^ | x^1^ |
|  | Right Orbitofrontal Cortex |  | x^2^ |  |  |  |  |  | Right Orbitofrontal Cortex | x^1^ | x^2^ |  | x^1^ | x^1^ |
|  | Right dACC |  | x^3^ |  |  |  |  |  | Left Middle Frontal Gyrus |  | x^1^ |  |  | x^1^ |
|  | Right dmPFC | x | x |  |  |  |  |  | Left Superior Frontal Gyrus |  | x |  |  |  |
|  | Left Middle Frontal Gyrus | x |  |  |  |  |  |  | Left Pars Triangularis |  | x |  |  |  |
|  | Right Frontal Pole | x |  |  |  |  |  |  | Left Pars Opercularis |  | x |  |  |  |
|  | Left pgACC | x |  |  |  |  |  |  |  |  |  |  |  |  |
| *Temporal* |  |  |  |  |  |  |  | *Temporal* |  |  |  |  |  |  |
|  | Right Middle Temporal Gyrus |  |  |  |  | x |  |  | - |  |  |  |  |  |
|  |  |  |  |  |  |  |  |  |  |  |  |  |  |  |
|  |  |  |  |  |  |  |  |  |  |  |  |  |  |  |
| *Parietal* |  |  |  |  |  |  |  | *Parietal* |  |  |  |  |  |  |
|  | Left Inferior Parietal Lobe | x |  |  |  |  |  |  | Left Postcentral Gyrus |  | x |  |  |  |
|  | Left Precuneus | x |  |  |  |  |  |  | Right Postcentral Gyrus |  | x |  |  | x^1^ |
|  | Left Angular Gyrus | x | x |  |  |  |  |  |  |  |  |  |  |  |
|  | Right Angular Gyrus | x | x |  |  |  |  |  |  |  |  |  |  |  |
| *Subcortical* |  |  |  |  |  |  |  | *Subcortical* |  |  |  |  |  |  |
|  | - |  |  |  |  |  |  |  | Left Thalamus | x^1^ | x^1^ |  | x^1^ | x^1^ |
|  |  |  |  |  |  |  |  |  | Right Thalamus | x^1^ | x^1^ |  | x^1^ | x^1^ |

*Note.* BLA = basolateral nuclei. CMA = centromedial nuclei. L = left hemisphere. R = right hemisphere. vm/dmPFC = ventromedial/dorsomedial prefrontal cortex. d/pgACC = dorsal/perigenual anterior cingulate cortex. No study investigated superficial nuclei connectivity. ^1^Results are common within the same studies. ^2^Results are contradictory across studies. ^3^Result obtained for multiple studies.

**Supplementary Table 2.**

*Table of Main Resting-State Functional Connectivity Results for Posttraumatic Stress Disorder (PTSD) vs. Non-Trauma-Exposed Controls (NEC)*

| **Contrast** | **Brain Region** | **BLA** | |  | **CMA** | |  | **Contrast** | **Brain Region** | **BLA** | |  | **CMA** | |  | **SFA** | |
| --- | --- | --- | --- | --- | --- | --- | --- | --- | --- | --- | --- | --- | --- | --- | --- | --- | --- |
|  |  | L | R |  | L | R |  |  |  | L | R |  | L | R |  | L | R |
| PTSD > NEC |  |  |  |  |  |  |  | PTSD < NEC |  |  |  |  |  |  |  |  |  |
| *Frontal* |  |  |  |  |  |  |  | *Frontal* |  |  |  |  |  |  |  |  |  |
|  | Left vmPFC |  | x |  |  |  |  |  | Right dACC |  | x |  |  |  |  |  |  |
|  | Left Orbitofrontal Cortex |  | x |  |  |  |  |  | Left Middle Frontal Gyrus |  |  |  |  | x |  |  |  |
|  | Right Orbitofrontal Cortex |  | x |  |  |  |  |  |  |  |  |  |  |  |  |  |  |
|  | Left Middle Frontal Gyrus | x |  |  |  |  |  |  |  |  |  |  |  |  |  |  |  |
|  | Right Middle Frontal Gyrus | x^1,2^ |  |  |  |  |  |  |  |  |  |  |  |  |  |  |  |
|  | Left Anterior Insula |  | x |  |  |  |  |  |  |  |  |  |  |  |  |  |  |
|  | Right Anterior Insula | x | x |  |  |  |  |  |  |  |  |  |  |  |  |  |  |
|  | Left Mid Insula | x^2^ |  |  |  |  |  |  |  |  |  |  |  |  |  |  |  |
|  | Right Mid Insula | x |  |  |  |  |  |  |  |  |  |  |  |  |  |  |  |
|  | Left Posterior Insula | x^2^ |  |  |  |  |  |  |  |  |  |  |  |  |  |  |  |
| *Temporal* |  |  |  |  |  |  |  | *Temporal* |  |  |  |  |  |  |  |  |  |
|  | Right Superior Temporal Gyrus |  |  |  |  | x |  |  | Left Superior Temporal Gyrus | x | x |  |  |  |  |  |  |
|  |  |  |  |  |  |  |  |  | Right Superior Temporal Gyrus |  | x |  |  |  |  |  |  |
|  |  |  |  |  |  |  |  |  | Right Fusiform Gyrus |  |  |  |  |  |  |  | x |
| *Occipital* |  |  |  |  |  |  |  | *Occipital* |  |  |  |  |  |  |  |  |  |
|  | Left Middle Occipital Gyrus |  |  |  |  | x |  |  | Left Middle Occipital Gyrus |  |  |  |  |  |  | x |  |
|  | Right Middle Occipital Gyrus |  |  |  |  | x |  |  | Left Lingual Gyrus |  |  |  |  |  |  | x |  |
| *Parietal* |  |  |  |  |  |  |  | *Parietal* |  |  |  |  |  |  |  |  |  |
|  | Left Angular Gyrus | x | x |  |  |  |  |  | Right Paracentral Gyrus |  | x |  |  |  |  |  |  |
|  | Right Angular Gyrus | x | x |  |  |  |  |  |  |  |  |  |  |  |  |  |  |
| *Subcortical* |  |  |  |  |  |  |  | *Subcortical* |  |  |  |  |  |  |  |  |  |
|  | Left Periaqueductal Grey | x |  |  |  |  |  |  | - |  |  |  |  |  |  |  |  |
|  | Right Periaqueductal Grey |  | x |  |  |  |  |  |  |  |  |  |  |  |  |  |  |

Note. BLA = basolateral nuclei. CMA = centromedial nuclei. SFA = superficial nuclei. L = left hemisphere. R = right hemisphere. vm/dmPFC = ventromedial/dorsomedial prefrontal cortex. d/pgACC = dorsal/perigenual anterior cingulate cortex. BA7 = superior parietal lobe/precuneus. BA28 = entorhinal cortex. ^1^Result obtained for multiple studies. ^2^Results also obtained for task-based study in HC.

**Supplementary Table 3.**

*Table of Main Task-Based Functional Connectivity Results for Posttraumatic Stress Disorder (PTSD) vs. Non-Trauma-Exposed Controls (NEC)*

| **Contrast** | **Brain Region** | **BLA** | |  | **CMA** | |  | **Contrast** | **Brain Region** | **BLA** | |  | **CMA** | |
| --- | --- | --- | --- | --- | --- | --- | --- | --- | --- | --- | --- | --- | --- | --- |
|  |  | L | R |  | L | R |  |  |  | L | R |  | L | R |
| PTSD > NEC |  |  |  |  |  |  |  | PTSD < NEC |  |  |  |  |  |  |
| *Frontal* |  |  |  |  |  |  |  | *Frontal* |  |  |  |  |  |  |
|  | Left dACC | x |  |  |  |  |  |  |  |  |  |  |  |  |
|  | Right dACC | x |  |  |  |  |  |  | - |  |  |  |  |  |
|  | Left Middle Frontal Gyrus | x |  |  |  |  |  |  |  |  |  |  |  |  |
|  | Right Middle Frontal Gyrus | x^1^ |  |  |  |  |  |  |  |  |  |  |  |  |
|  | Right Superior Frontal Gyrus |  |  |  |  | x |  |  |  |  |  |  |  |  |
|  | Right Medial Frontal Gyrus | x |  |  |  |  |  |  |  |  |  |  |  |  |
|  | Left Insula | x^1^ |  |  |  |  |  |  |  |  |  |  |  |  |
|  | Right Insula | x |  |  |  |  |  |  |  |  |  |  |  |  |
| *Temporal* |  |  |  |  |  |  |  | *Temporal* |  |  |  |  |  |  |
|  | Right Inferior Temporal Gyrus | x |  |  |  |  |  |  | Right Middle Temporal Gyrus | x^2^ |  |  |  |  |
|  | Right Middle Temporal Gyrus | x^1^ |  |  |  |  |  |  |  |  |  |  |  |  |
|  | Right Superior Temporal Gyrus | x |  |  |  |  |  |  |  |  |  |  |  |  |
| *Parietal* |  |  |  |  |  |  |  | *Parietal* |  |  |  |  |  |  |
|  | Left Inferior Parietal Lobe | x |  |  |  |  |  |  | Right Postcentral Gyrus | x |  |  |  |  |
|  |  |  |  |  |  |  |  |  | Right BA7 | x |  |  |  |  |
| *Subcortical* |  |  |  |  |  |  |  | *Subcortical* |  |  |  |  |  |  |
|  | Left Hippocampus | x |  |  |  |  |  |  | Right Superior Colliculus |  | x |  |  |  |
|  | Right Parahippocampal Gyrus | x |  |  |  |  |  |  | Left Brainstem | x^2^ |  |  |  |  |
|  | Left BA28 | x |  |  |  |  |  |  |  |  |  |  |  |  |
|  | Right BA28 | x |  |  |  |  |  |  |  |  |  |  |  |  |
|  | Left Pulvinar |  |  |  | x |  |  |  |  |  |  |  |  |  |
|  | Left Brainstem | x^1^ |  |  |  |  |  |  |  |  |  |  |  |  |

*Note.* BLA = basolateral nuclei. CMA = centromedial nuclei. L = left hemisphere. R = right hemisphere. vm/dmPFC = ventromedial/dorsomedial prefrontal cortex. d/pgACC = dorsal/perigenual anterior cingulate cortex. BA7 = approximately, superior parietal lobe. BA28 = approximately, entorhinal cortex. ^1^Results also obtained for resting-state study in healthy controls. ^2^Result contradictory within the same study.

**Supplementary Figure 1.**

*Task-Based Amygdala Subnuclei Connectivity in Posttraumatic Stress Disorder (PTSD) vs. Non-Trauma-Exposed Controls (NEC)*

**
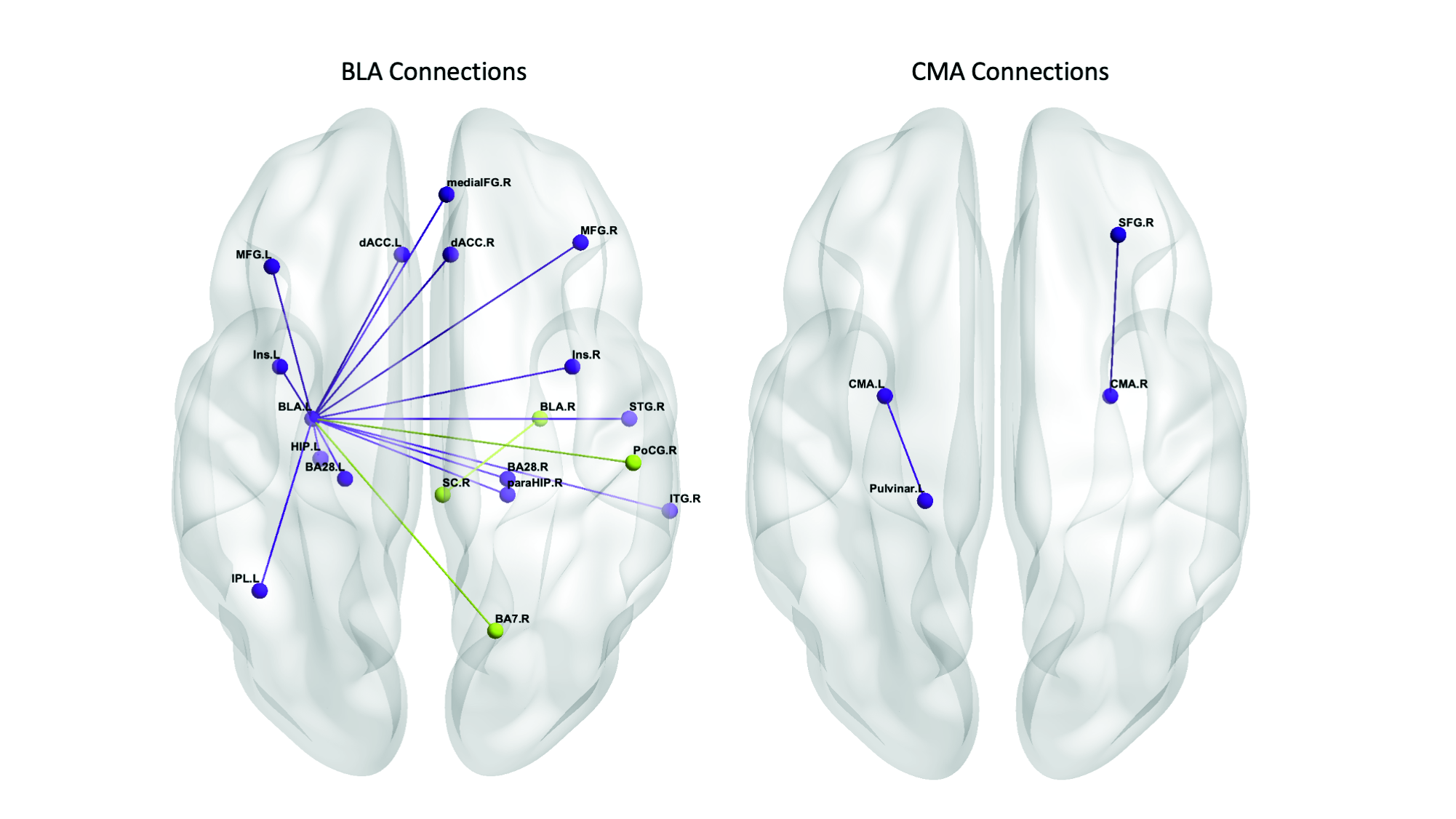
**

*Note.* Basolateral subnuclei connections on the left, centromedial subnuclei connections on the right. Greater connectivity for PTSD vs. NEC is signified by dark/purple colours; lesser connectivity for PTSD vs. NEC is signified by light/green colours. Image orientation is neurological—left hemisphere on the left, frontal lobe at the top. PTSD = Posttraumatic stress disorder. NEC = Non-trauma-exposed controls. L = left. R = right. BLA = basolateral amygdala nucleus. CMA = centromedial amygdala nucleus. medialFG = medial frontal gyrus. dACC = dorsal anterior cingulate cortex. SFG = superior frontal gyrus. Ins = insula. PoCG = postcentral gyrus. I/M/STG = inferior/middle/superior temporal gyrus. BA7 = approximately, superior parietal lobe. HIP = hippocampus. BA28 = approximately, entorhinal cortex. paraHIP = parahippocampal gyrus. SC = superior colliculus.
